# Lung Recruitment Before Surfactant Administration in Extremely Preterm Neonates: 2-Year Follow-Up of a Randomized Clinical Trial

**DOI:** 10.1001/jamanetworkopen.2024.35347

**Published:** 2024-09-25

**Authors:** Francesca Gallini, Domenico Umberto De Rose, Roberta Iuliano, Domenico Marco Romeo, Milena Tana, Angela Paladini, Francesca Paola Fusco, Stefano Nobile, Francesco Cota, Chiara Tirone, Claudia Aurilia, Alessandra Lio, Alice Esposito, Simonetta Costa, Vito D’Andrea, Maria Luisa Ventura, Virgilio Carnielli, Carlo Dani, Fabio Mosca, Monica Fumagalli, Gianfranco Scarpelli, Lucio Giordano, Valeria Fasolato, Flavia Petrillo, Pasqua Betta, Agostina Solinas, Eloisa Gitto, Giancarlo Gargano, Giovanna Mescoli, Stefano Martinelli, Sandra Di Fabio, Italo Bernardo, Lucia Gabriella Tina, Alex Staffler, Ilaria Stasi, Isabella Mondello, Eleonora Scapillati, Stefania Vedovato, Gianfranco Maffei, Adriano Bove, Marcello Vitaliti, Gianluca Terrin, Paola Lago, Camilla Gizzi, Chiara Strozzi, Paolo Ernesto Villani, Alberto Berardi, Caterina Cacace, Giorgio Bracaglia, Eleonora Pascucci, Filip Cools, Jane J. Pillow, Graeme Polglase, Roberta Pastorino, Anton H. van Kaam, Eugenio Mercuri, Luigi Orfeo, Giovanni Vento, and the IN-REC-SUR-E Study Group, Silvia Malguzzi, Camilla Rigotti, Alessandra Cecchi, Gabriella Nigro, Carmine Deni Costabile, Enza Roma, Paola Sindico, Rita Venafra, Carmine Mattia, Maria Conversano, Elisa Ballardini, Alessandro Manganaro, Eleonora Balestri, Claudio Gallo, Piero Catenazzi, Maria Graziana Astori, Eugenia Maranella, Carolina Grassia, Kim Maiolo, Danilo Castellano, Luca Massenzi, Elisabetta Chiodin, Maria Rita Gallina, Chiara Consigli, Elena Sorrentino, Silvia Bonato, Monica Mancini, Roberto Perniola, Silvia Giannuzzo, Elisa Tranchina, Viviana Cardilli, Lucia Dito, Daniela Regoli, Francesca Tormena, Nadia Battajon, Roberta Arena, Benedetta Allais, Isotta Guidotti, Federica Roversi, Valerio Meli, Viviana Tulino, Alessandra Casati

**Affiliations:** 1 Neonatology Unit, Department of Woman and Child Health and Public Health, Fondazione Policlinico Universitario Agostino Gemelli Istituto di Ricovero e Cura a Carattere Scientifico, Rome, Italy; 2 Dipartimento di Scienze della Vita e Sanità Pubblica, Facoltà di Medicina e Chirurgia, Università Cattolica del Sacro Cuore, Rome, Italy; 3 Neonatology Unit, Ospedale Isola Tiberina, Gemelli Isola, Rome, Italy; 4 Neonatal Intensive Care Unit, Bambino Gesù Children’s Hospital Istituto di Ricovero e Cura a Carattere Scientifico, Rome, Italy; 5 Pediatric Neurology Unit, Fondazione Policlinico Universitario Agostino Gemelli Istituto di Ricovero e Cura a Carattere Scientifico, Rome, Italy; 6 Fondazione Monza e Brianza per il Bambino e la sua Mamma, Ospedale San Gerardo, Monza, Italy; 7 Division of Neonatology, Department of Clinical Sciences, Polytechnic University of Marche and Azienda Ospedaliero Universitaria Ospedali Riuniti, Ancona, Italy; 8 Department of Mother and Child Health, Division of Neonatology and Neonatal Intensive Care Unit, Careggi University Hospital, Florence, Italy; 9 Department of Clinical Sciences and Community Health, University of Milan, Fondazione Istituto di Ricovero e Cura a Carattere Scientifico Cà Granda Ospedale Maggiore Policlinico, Milan, Italy; 10 Azienda Ospedaliera Cosenza, Cosenza, Italy; 11 Ospedale Pineta Grande, Castel Volturno, Italy; 12 Azienda Ospedaliera Carlo Poma, Mantova, Italy; 13 Dipartimento Materno Infantile ASL Bari, Ospedale Di Venere, Bari, Italy; 14 Azienda Ospedaliera-Universitaria Policlinico Vittorio Emanuele, Presidio Ospedaliero Gaspare Rodolico, Catania, Italy; 15 Azienda Ospedaliera-Universitaria, Ferrara, Italy; 16 Università degli Studi, Messina, Italy; 17 Azienda Unità Sanitaria Locale, Istituto di Ricovero e Cura a Carattere Scientifico, Reggio Emilia, Italy; 18 Ospedale Maggiore, Bologna, Italy; 19 Azienda Socio Sanitaria Territoriale Grande Ospedale Metropolitano Niguarda, Milan, Italy; 20 Ospedale San Salvatore, L’Aquila, Italy; 21 Azienda Ospedaliera S. Anna e S. Sebastiano, Caserta, Italy; 22 Azienda ospedaliera di rilievo nazionale e di alta specializzazione Garibaldi, Catania, Italy; 23 Ospedale di Bolzano, Bolzano, Italy; 24 Ospedale Maggiore, Novara, Italy; 25 Azienda Ospedaliera Bianchi-Melacrino-Morelli, Reggio Calabria, Italy; 26 Ospedale Fatebenefratelli-San Pietro, Rome, Italy; 27 Ospedale San Bortolo, Vicenza, Italy; 28 Azienda Ospedaliera-Universitaria Ospedali Riuniti, Foggia, Italy; 29 Azienda Ospedaliera Vito Fazzi, Lecce, Italy; 30 Azienda ospedaliera di rilievo nazionale e di alta specializzazione Civico, Palermo, Italy; 31 Maternal and Child Health Department, University of Rome Sapienza, Rome, Italy; 32 Ospedale Cà Foncello, Treviso, Italy; 33 Ospedale Sandro Pertini, Rome, Italy; 34 Ospedale Civile SS. Antonio e Biagio e Cesare Arrigo, Alessandria, Italy; 35 Fondazione Poliambulanza, Brescia, Italy; 36 Azienda Ospedaliera-Universitaria Policlinico, Modena, Italy; 37 Ospedale Barone Romeo, Patti, Italy; 38 Ospedale Belcolle, Viterbo, Italy; 39 Department of Neonatology, Universitair Ziekenhuis Brussel, Brussels, Belgium; 40 Centre for Child Health Research and School of Human Sciences, The University of Western Australia, Perth, Australia; 41 The Ritchie Centre Hudson Institute of Medical Research and Department of Obstetrics and Gynaecology, Monash University, Clayton, Australia; 42 Section of Hygiene, Department of Life Sciences and Public Health, Università Cattolica del Sacro Cuore, Rome, Italy; 43 Department of Woman and Child Health and Public Health–Public Health Area, Fondazione Policlinico Universitario A. Gemelli Istituto di Ricovero e Cura a Carattere Scientifico, Rome, Italy; 44 Department of Neonatology, Emma Children’s Hospital, Amsterdam University Medical Center, University of Amsterdam, Vrije Universiteit Amsterdam, Amsterdam, the Netherlands

## Abstract

**Importance:**

A multicenter randomized clinical trial (RCT) showed a lung recruitment maneuver using high-frequency oscillatory ventilation just before surfactant administration (ie, intubate-recruit-surfactant-extubate [IN-REC-SUR-E]) improved the efficacy of treatment compared with the standard intubate-surfactant-extubate (IN-SUR-E) technique without increasing the risk of adverse neonatal outcomes.

**Objective:**

To examine follow-up outcomes at corrected postnatal age (cPNA) 2 years of preterm infants previously enrolled in an RCT and treated with IN-REC-SUR-E or IN-SUR-E in 35 tertiary neonatal intensive care units.

**Design, Setting, and Participants:**

This was a follow-up study of infants recruited into the primary RCT from 2015 to 2018 at 35 tertiary neonatal intensive care units (NICUs) in Italy. Follow-up examinations included neurodevelopmental, growth, and respiratory outcomes of these children at cPNA 2 years. Participants included spontaneously breathing extremely preterm neonates (24 0/7 to 27 6/7 weeks’ gestation) reaching failure criteria for continuous positive airway pressure within the first 24 hours of life. Data were analyzed from April 2023 to January 2024.

**Intervention:**

Infants were randomly assigned (1:1) to IN-REC-SUR-E or IN-SUR-E and then followed up.

**Main Outcomes and Measures:**

The primary outcome was the occurrence of death after discharge or major disability at cPNA 2 years. Secondary outcomes were neurodevelopmental outcomes (major disability, cerebral palsy, cognitive impairment, visual deficit, or auditory deficit), anthropometric measurements (weight, length, and head circumference), and recurrent respiratory infections and hospitalizations because of respiratory causes at 2y cPNA.

**Results:**

A total of 137 extremely preterm infants (median [IQR] gestational age, 26.5 [25.3-27.5] weeks and 75 [54.7%] female), initially enrolled in the original RCT, were followed up at cPNA 2 years, including 64 infants in the IN-SUR-E group and 73 infants in the IN-REC-SUR-E group. There were no significant differences in the occurrence of death after discharge or major disability at cPNA 2 years (IN-SUR-E: 13 children [20.3%] vs IN-REC-SUR-E: 10 children [13.7%]; *P* = .36). There were no significant differences in incidence of disability, cerebral palsy, or cognitive impairment in the IN-REC-SUR-E group compared with the IN-SUR-E group. There were no significant differences in anthropometric measurements (weight, length, and head circumference) between groups. There were no significant differences in the incidence of recurrent respiratory infections or in hospitalizations because of respiratory causes between groups.

**Conclusions and Relevance:**

In this RCT of lung recruitment before surfactant vs standard care there were no significant differences between the 2 groups in death, neurodevelopmental outcomes, anthropometric measurements, or recurrent respiratory infections at the 2-year follow-up. These findings can aid clinicians in decision-making for the best strategy to administer surfactant, considering long-term outcomes.

## Key Points

**Question:**

Did infants who underwent the intubate-recruit-surfactant-extubate lung recruitment technique, compared with those treated with the intubate-surfactant-extubate technique, have better outcomes at the 2-year follow-up?

**Findings:**

In this randomized clinical trial including 131 infants at the 2-year follow-up, no significant differences were found in the occurrence of death after discharge or major disability. There were also no significant differences in incidence of major disability, cerebral palsy, or cognitive impairment.

**Meaning:**

These findings suggest that the intubate-recruit-surfactant-extubate technique is a safe method for the measured outcomes.

## Teaser

This randomized clinical trial follows up extremely preterm infants treated at birth with a high-frequency oscillatory ventilation just before surfactant administration technique vs the standard intubate-surfactant-extubate technique to examine outcomes at corrected postnatal age 2 years.

## Introduction

Morbidity and mortality associated with prematurity inversely correlate to gestational age (GA) at birth, with each additional week conferring survival benefit.^1^ Extremely low gestational age newborns (GA <28 weeks) have a higher risk for neonatal respiratory distress syndrome requiring prolonged mechanical ventilation (MV), and subsequent development of bronchopulmonary dysplasia (BPD).^2^

We recently showed that a lung recruitment maneuver using high-frequency oscillatory ventilation (HFOV) just before surfactant administration (intubate-recruit-surfactant-extubate [IN-REC-SUR-E]) reduces the need for MV in the first 72 hours of life compared with the standard IN-SUR-E technique in extremely preterm neonates, without increasing the risk of adverse neonatal outcomes. This reduction in MV may make applying a noninvasive respiratory support strategy easier.^3^

Regardless of severity, infants with BPD can have worse neurodevelopmental outcomes than their peers without BPD.^4,5^ Therefore, it is crucial to know whether an initial strategy reducing MV will influence long-term outcomes, helping clinicians to choose the more advantageous surfactant administration technique.

We hypothesized that infants assigned to the IN-REC-SUR-E group would have improved neurodevelopmental, growth, and respiratory outcomes compared with the IN-SUR-E group. In this study, we report the neurodevelopmental, growth, and respiratory outcomes at corrected postnatal age (cPNA) 2 years of the infants included in the IN-REC-SUR-E study.

## Methods

This randomized clinical trial was approved by the human research ethics committee of the Fondazione Policlinico Universitario Agostino Gemelli Istituto di Ricovero e Cura a Carattere Scientifico. Written informed consent was obtained from parents before inclusion in the study. The trial protocol and statistical analysis plan are provided in Supplement 1. This study is reported following the Consolidated Standards of Reporting Trials (CONSORT) reporting guideline.

### Study Design

This is a 2-year follow-up of infants enrolled in a multicenter, unblinded, controlled, randomized clinical trial previously conducted in 35 tertiary neonatal intensive care units (NICUs) in Italy.^3^ Enrolled patients were recruited from November 12, 2015, to September 23, 2018. Infants were eligible for the study if they were born in a tertiary NICU participating in the trial, had a GA of 24 0/7 to 27 6/7 weeks, were breathing independently with only nasal continuous positive airway pressure for respiratory support, and met surfactant administration criteria during the first 24 hours of life. Infants were ineligible if they had severe birth asphyxia or a 5-minute Apgar score less than 3, required endotracheal intubation in the delivery room for resuscitation or insufficient respiratory drive, were born after prolonged (>21 days) premature rupture of membranes, or had a major congenital abnormality, inherited disorder of metabolism, or hydrops fetalis.

Infants were randomized 1:1 to 1 of 2 treatment groups: IN-REC-SUR-E (intervention group) or IN-SUR-E (control group), using an interactive web-based electronic system according to the minimization method. After NICU discharge, all 156 surviving infants were enrolled in a 2-year anthropometric and neurodevelopmental follow-up program.

### Outcomes

The primary outcome was the occurrence of death after discharge or major disability at cPNA 2 years. The secondary outcomes were neurodevelopmental outcomes (major disability, cerebral palsy, cognitive impairment, visual deficit, or auditory deficit), anthropometric measurements (weight, length, and head circumference), and recurrent respiratory infections and hospitalizations because of respiratory causes at cPNA 2 years.

### Follow-Up and Definitions

Clinical follow-up visits included a standardized neurodevelopmental, growth, and respiratory assessment.^6^ The long-term neurodevelopmental outcome was evaluated at cPNA 2 years using Griffith’s Mental Developmental Scales (GMDS) (ages 0-2 years)^7^ or Bayley Development Scale for Toddlers and Infants, Third Edition (BSDI-III),^8^ according to the experience of each participating center. The assessors at follow-up were blinded to the treatment allocation of the neonatal intervention (IN-REC-SUR-E vs IN-SUR-E).

Cognitive outcome was classified as within reference range when developmental quotient (DQ) was greater than 85, borderline when DQ was from 70 to 85, and delayed when DQ was lower than 70. Major disability was defined as the presence of at least 1 among the following: cerebral palsy (according to Bax classification^9^), cognitive impairment (defined by a DQ <70), visual impairment (visual acuity <6/60 in the better eye),^10^ and hearing impairment (ie, deafness requiring bilateral hearing aids or unilateral or bilateral cochlear implants).^11^

The anthropometric evaluation was performed by assessing the infant’s weight using electronic scales, while length was determined using the Harpenden neonatometer or a fixed stadiometer evaluating the crown-heel measurement. Head circumference was determined by measuring the maximal occipital-frontal circumference through a centimeter tape of inextensible material. These values were converted to the sex- and age-specific *z* score of weight, height, and head circumference at cPNA 2 years using the World Health Organization Anthro software version 3.2.2, based on the World Health Organization Child Growth Standards 2006. Furthermore, we collected information on the presence of recurrent respiratory pathology (defined as ≥2 episodes per year of wheezing, infections, or both) and hospitalizations in the first 2 years of life via a specifically designed questionnaire (not validated).

### Statistical Analysis

All data were retrospectively collected and stored on a dedicated database. Data are presented as numbers and percentages for categorical variables. Continuous variables are expressed as mean and SD if they were normally distributed or as the median and IQR for nonnormal data. Comparisons between continuous variables were performed using the *t* test in the case of normal distribution and the Mann-Whitney *U* test in the case of nonnormal distribution. Categorical variables were compared using Fisher exact test or χ^2^ with Yates correction.

Treatment group and clinical characteristics (variables that were significant in the univariate analysis: birth weight, the need for MV in the first 72 hours of life, and intraventricular hemorrhage grade >2) were included in a multivariable log-binomial regression analysis to assess their independent associations with the primary outcome. Effect estimates were expressed as odds ratios (ORs) with 95% CIs. No adjustments for multiple comparisons were made. Hence, these analyses should be interpreted as exploratory.

A 2-tailed *P* < .05 was considered significant. Statistical analysis was performed using Stata software version 15.1 for Windows (StataCorp). Data were analyzed from April 2023 to January 2024.

## Results

### Study Population

A total of 218 preterm infants were enrolled in the original RCT.^3^ The primary outcome was evaluated in 137 children, whereas secondary long-term outcomes were evaluated at cPNA 2 years in a final population of 137 children (median [IQR] gestational age, 26.5 [25.3-27.5] weeks and 75 [54.7%] female): 64 from the IN-SUR-E control group and 73 from the IN-REC-SUR-E intervention group (Figure).

**Figure.**
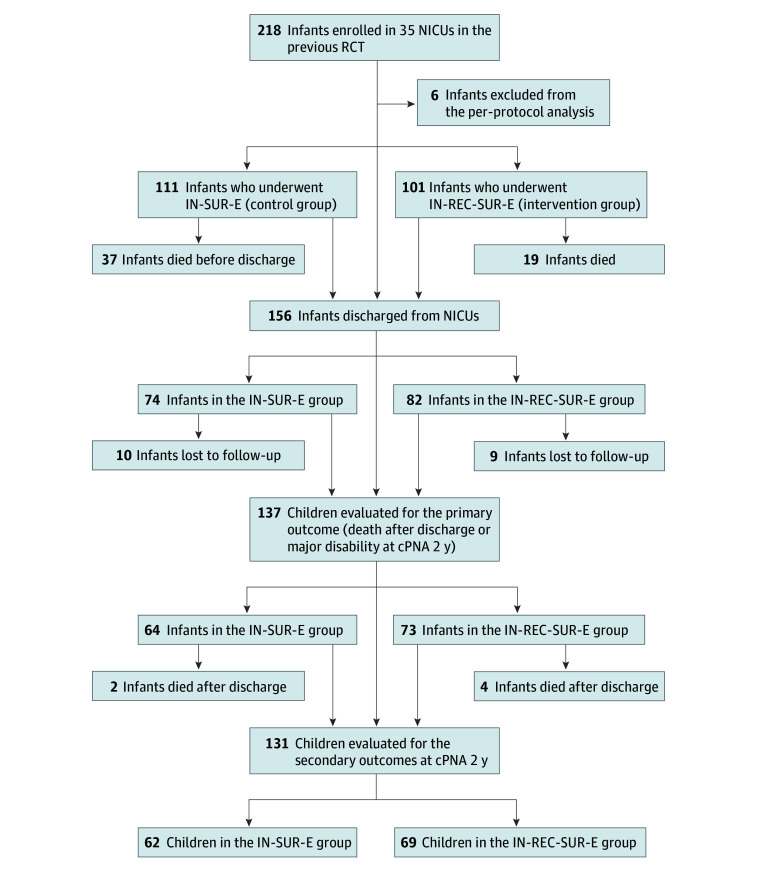
Flowchart of Randomization, Enrollment, and Follow-Up

Prior to this follow-up study, of 212 infants in the per-protocol analysis, 37 of 111 infants (33.3%) in the IN-SUR-E group and 19 of 101 infants (18.8%) in the IN-REC-SUR-E group died during the NICU stay (*P* = .02). A total of 156 infants were then discharged alive from involved NICUs and enrolled in a follow-up program. Of these, 10 of 74 infants (13.5%) from the IN-SUR-E group and 9 of 82 infants (11.0%) from the IN-REC-SUR-E group were lost during follow-up (*P* = .63) (Figure).

During the first months of the follow-up period, further 2 of 64 infants (3.1%) from the IN-SUR-E group died (1 from bronchiolitis and 1 from sepsis) and 4 of 73 infants (5.5%) from the IN-REC-SUR-E group died (1 from hypovolemic shock during gastroenteritis, 1 from pneumonia, 1 from respiratory failure in severe BPD, and 1 from bronchiolitis) died (*P* = .68). Overall mortality (including NICU stay and the follow-up period) reached 35.1% in the IN-SUR-E group (39 infants) and 22.8% in the IN-REC-SUR-E group (23 infants) (*P* = .05).

The baseline characteristics of the 2 study groups at the 2-year follow-up were representative of the initial RCT neonatal population, with no significant differences, except for vaginal delivery (Table 1). In addition, no statistically significant differences were found in the respiratory support received: prior to surfactant administration, 45 IN-REC-SUR-E infants (65.2%) and 34 IN-SUR-E infants (54.8%) required 1 sustained lung inflation maneuver (*P* = .23); 2 sustained lung inflation maneuvers were needed in 18 IN-REC-SUR-E infants (26.1%) and 24 IN-SUR-E infants (38.7%) (*P* = .12). Prior to surfactant administration, the median (IQR) continuous positive airway pressure values were 6.0 (6.0-6.5) cm H_2_O in the IN-REC-SUR-E group vs 6 (6.0-6.5) cm H_2_O in the IN-SUR-E group (*P* = .86) and the median (IQR) fraction of inspired oxygen was 0.30 (0.28-0.38) in the IN-REC-SUR-E group vs 0.30 (0.26-0.40) in the IN-SUR-E group (*P* = .62).

**Table 1.** Baseline Demographics of Included Infants at the 2-Year Follow-Up

Characteristic	Infants, No. (%) (N = 131)		*P* value
	IN-SUR-E (n = 62)	IN-REC-SUR-E (n = 69)	
GA, wk			
Median (IQR)	26.86 (25.71-27.29)	26.29 (25.86-27.43)	.59
24-25	18 (29.0)	21 (30.4)	.86
26-27	44 (71.0)	48 (69.6)	.86
Birth weight			
Mean (SD), g	819.82 (192.97)	873.54 (189.53)	.11
<10° centile	9 (14.5)	4 (5.8)	.14
<3° centile	2 (3.2)	1 (1.5)	.60
Sex			
Female	34 (54.8)	38 (55.1)	.98
Male	28 (45.2)	31 (44.9)	
Antenatal steroids	56 (90.3)	67 (97.1)	.15
Vaginal delivery	8 (12.9)	19 (27.5)	.04
Preterm premature rupture of membranes	21 (33.9)	17 (24.6)	.25
Chorioamnionitis	9 (14.5)	8 (11.6)	.62

Abbreviations: GA, gestational age; IN-REC-SUR-E, intubate-recruit-surfactant-extubate; IN-SUR-E, intubate-surfactant-extubate.

Finally, similarly to the results of the original trial, the requirement for MV during the first 72 hours of life (initial primary outcome) was significantly reduced in the IN-REC-SUR-E group (16 infants [23.2%]) compared with the IN-SUR-E group (30 infants [48.4%]) (*P* = .003). Table 2 shows the other neonatal characteristics in included children assessed at follow-up. The mean (SD) cPNA at follow-up evaluation was comparable between groups, with 25.3 (7.2) months in the IN-SUR-E group vs 24.6 (6.4) months in the IN-REC-SUR-E group (*P* = .55).

**Table 2.** Neonatal Characteristics of Included Infants at the 2-Year Follow-Up

Characteristic	Infants, No. (%) (N = 131)		*P* value
	IN-SUR-E (n = 62)	IN-REC-SUR-E (n = 69)	
Mechanical ventilation during the first 72 h of life	30 (48.4)	16 (23.2)	.003
>1 Dose surfactant	29 (46.8)	25 (36.2)	.22
Invasive respiratory support, median (IQR), d	8 (4-21)	9 (0-25)	.79
Noninvasive respiratory support, median (IQR), d	39 (28-54)	40 (27.5-53)	.80
Oxygen therapy, median (IQR), d	32.5 (14-54)	39.5 (10-61.5)	.89
Moderate to severe bronchopulmonary dysplasia	46 (75.4)	45 (65.2)	.21
Pneumothorax	3 (4.8)	1 (1.5)	.34
Pulmonary interstitial emphysema	4 (6.6)	4 (5.9)	>.99
Hemodynamically significant patent ductus arteriosus	5 (8.3)	7 (10.1)	.72
Pulmonary hemorrhage	2 (3.2)	3 (4.4)	>.99
Intraventricular hemorrhage grade >2	4 (6.5)	6 (8.7)	.75
Periventricular leukomalacia	2 (3.2)	8 (11.6)	.10
Sepsis	36 (58.1)	40 (58.0)	.99
Necrotizing enterocolitis	5 (8.1)	5 (7.3)	.86
Retinopathy of prematurity grade >2	11 (17.7)	13 (18.8)	.87
Postnatal steroids	24 (38.7)	25 (36.2)	.77
Length of stay, median (IQR), d	92 (77-119)	94 (74-112)	.90

Abbreviations: IN-REC-SUR-E, intubate-recruit-surfactant-extubate; IN-SUR-E, intubate-surfactant-extubate.

### Primary Outcome

We found no significant differences in the occurrence of death after discharge or major disability at cPNA 2 years. Of 64 children in the IN-SUR-E group, 13 died or had a major disability (20.3%), compared with 10 of 73 children in the IN-REC-SUR-E group (13.7%) (*P* = .36) (Table 3).

**Table 3.** Neurodevelopmental Outcomes at the 2-Year Follow-Up

Outcome	Infants, No. (%) (N = 131)		*P* value
	IN-SUR-E (n = 62)	IN-REC-SUR-E (n = 69)	
Major disability	11 (17.7)	6 (8.7)	.19
Cerebral palsy	7 (11.3)	2 (2.9)	.08
Cognitive impairment	10 (16.1)	5 (7.2)	.17
Visual deficit	1 (1.6)	2 (2.9)	>.99
Auditory deficit	0	0	NA

Abbreviations: IN-REC-SUR-E, intubate-recruit-surfactant-extubate; IN-SUR-E, intubate-surfactant-extubate; NA, not applicable.

### Secondary Outcomes

#### Long-Term Neurodevelopmental Outcomes

Long-term neurodevelopmental outcomes were globally evaluated in all included infants, and there were no significant differences in neurodevelopmental outcomes between groups (Table 3). A subanalysis was performed considering only the developmental quotient (DQ) from GMDS or BSDI-III evaluation, but this specific evaluation was not available in all infants due to the study’s retrospective design and the peculiar timeframe of the overlapping SARS-CoV-2 pandemic. The evaluation of DQ was available for 54 of 69 children (78.3%) in the IN-REC-SUR-E intervention group and 47 of 62 children (75.8%) in the IN-SUR-E control group (*P* = .84). The mean (SD) DQ was 86.2 (16.2) in the IN-SUR-E group and 90.7 (14.9) in the IN-REC-SUR-E group (*P* = .16) (eFigure in Supplement 2). There was no significant difference in delayed neurodevelopment (DQ <70) between children in the IN-REC-SUR-E group (5 of 54 children [9.3%]) compared with the children in the IN-SUR-E group (10 of 47 children [20.5%]) (*P* = .16). There was also no significant difference in borderline or delayed neurodevelopment (DQ <85) between children in the IN-REC-SUR-E group (14 of 52 children [26.9%]) compared with the children in the IN-SUR-E group (17 of 44 children [38.6%]) (*P* = .28).

#### Long-Term Growth and Respiratory Outcomes

Anthropometric measurements (weight, length, and head circumference) were not significantly different between groups at cPNA 2 years (Table 3). An analysis of the distribution of weights, heights, and head circumferences below the third percentile (*z* score ≤−1.88) at cPNA 2 years found no significant differences between groups (Table 4).

**Table 4.** Growth Outcomes at the 2-Year Follow-Up

Outcome	Infants (n = 92)		*P* value
	IN-SUR-E (n = 42)	IN-REC-SUR-E (n = 50)	
Weight			
Median (IQR), g	10 575 (10 000 to 12 000)	11 325 (10 000 to 12 000)	.44
*Z* score, mean (SD)	−1.0 (1.5)	−0.5 (1.2)	.14
Height			
Median (IQR), cm	83.5 (81 to 87.5)	84.7 (82 to 88)	.43
*Z* score, mean (SD)	−0.9 (1.3)	−0.6 (1.2)	.34
Head circumference			
Median (IQR), cm	47.25 (45.5 to 49)	47 (46 to 48.5)	.71
*Z* score, mean (SD)	−0.3 (1.6)	−0.2 (1.3)	.65

Abbreviations: IN-REC-SUR-E, intubate-recruit-surfactant-extubate; IN-SUR-E, intubate-surfactant-extubate.

Furthermore, considering the available data for respiratory follow-up (not available in all infants), the incidence of recurrent respiratory infections was not different between groups (IN-SUR-E: 16 of 47 children [34.0%]; IN-REC-SUR-E: 16 of 52 children [30.8%]; *P* = .73). Most patients received a complete prophylaxis with palivizumab against the respiratory syncytial virus (IN-SUR-E: 40 of 44 children [90.9%]; IN-REC-SUR-E: 47 of 53 children [88.7%]; *P* = .72).

No differences were found in the percentage of children attending kindergarten in the first 2 years of life (IN-SUR-E: 21 of 47 children [44.7%]; IN-REC-SUR-E: 20 of 51 children [39.2%]; *P* = .58). Similarly, in the same period, 9 of 47 children (19.15%) were hospitalized because of respiratory causes at least once in the IN-SUR-E group, while 10 of 52 children (19.2%) were admitted in the IN-REC-SUR-E group (*P* = .99).

#### Multivariable Log-Binomial Regression Analysis

The results of the multivariable log-binomial regression model indicated that birth weight and intraventricular hemorrhage greater than grade 2 were statistically significantly associated with the occurrence of death after discharge or major disability at cPNA 2 years. Birth weight was associated with lower odds of death or major disability (OR, 0.74; 95% CI, 0.56-0.96; *P* = .03), and intraventricular hemorrhage greater than grade 2 was associated with higher odds (OR, 3.42; 95% CI, 1.13-10.66; *P* = .04). Conversely, the need for MV in the first 72 hours was not associated with the primary outcome. The type of treatment did not affect the primary outcome. Despite the lack of significance in the type of treatment, it was observed that the IN-REC-SUR-E treatment reduced the probability of major disabilities or death after discharge at 2 years cPNA by 16%.

## Discussion

This RCT evaluated the follow-up of extremely preterm infants with respiratory distress syndrome enrolled in a previous RCT and randomized to receive IN-REC-SUR-E or the traditional IN-SUR-E. Our multicenter RCT demonstrated the efficacy of this novel strategy aimed at reducing MV in the early hours of life in neonates with respiratory distress syndrome, introducing a lung recruitment maneuver via HFOV before surfactant administration. The failure to find a significant reduction in the prevalence of moderate to severe forms of BPD in the IN-REC-SUR-E group was explained by the unexpectedly lower in-hospital mortality in the IN-REC-SUR-E group than in the IN-SUR-E group, mainly evident in the per-protocol analysis.^3^

During follow-up, we found no significant differences in the occurrence of death after discharge or major disability at cPNA 2 years (19.4% in the IN-SUR-E group vs 14.5% in the IN-REC-SUR-E group). Analyzing the causes of death after discharge, 2 infants died from respiratory complications related to severe bronchiolitis (1 in the IN-SUR-E group and 1 in the IN-REC-SUR-E group), 2 other infants died from infectious diseases (1 from sepsis in the IN-SUR-E group and 1 due to hypovolemic shock secondary to gastroenteritis in the IN-REC-SUR-E group). The final 2 infants in the IN-REC-SUR-E group died from pneumonia (1 infant) and severe BPD (1 infant). Based on these data, even if the protective effect of the lung recruitment procedure on death was lost in the postdischarge period, compared with the NICU stay, the causes of death appeared to depend mainly on complications related to prematurity per se.

Previously, most trials about elective HFOV vs conventional ventilation for acute pulmonary dysfunction in preterm infants did not find significant differences in the long-term neurodevelopmental outcomes of survivors,^12^ although a study by Sun et al^13^ showed a significant reduction in the risk of cerebral palsy and poor mental development in the HFOV group. Our cohort similarly showed that the IN-REC-SUR-E maneuver did not significantly influence neurodevelopmental outcomes at cPNA 2 years, despite an improved respiratory trend and a reduced need for MV in the first 72 hours of life.^3^ Long-term neurodevelopmental outcomes were similar between groups in terms of overall disability, either evaluated as the prevalence of infants with major disabilities (including single domains of disability) or as the prevalence of infants with reference range, borderline, and pathologic DQs.

Beyond mortality, children born at extremely low GA are known to experience higher morbidity rates, especially in the neuropsychological domain.^14,15^ The incidence of disability in our population can thus be considered consistent with the literature available on long-term outcomes of comparable populations.^16,17,18^ The mean DQ distribution was not statistically different between groups, although fewer children in the IN-REC-SUR-E group showed evidence of delayed neurodevelopment compared with the IN-SUR-E group. Two different tools (GMDS and BSDI-III) were used to assess DQ in recruiting centers: it is unlikely that using GMDS and BSDI-III influenced the results, given the excellent agreement between the scales previously reported in the evaluation of the neurodevelopmental outcome of extremely low birth weight infants at 24 months’ corrected age.^19^

These findings can be considered further evidence of the safety of this new technique on the long-term neurodevelopment of children with respect to the standard IN-SUR-E, although the number of infants lost to follow-up should be considered. Similarly, growth was not affected by the intervention, as no significant differences emerged between groups at follow-up in any of the studied anthropometric parameters. We found no significant differences in the rate of hospitalizations for respiratory causes between groups.

### Limitations

This study has some limitations. As our RCT was not originally designed to include a follow-up protocol, no a priori estimate of composite death or major disability was made. Considering the peculiar timeframe of the overlapping SARS-CoV-2 pandemic, the results of hospitalization rates need to be cautiously interpreted, since hospital admission criteria for preterm infants in the first 2 years of life could vary greatly from 1 hospital to another. For this reason, we believe these results cannot be linked with certainty to the different treatments that children initially received. As the RCT was not originally designed to include a follow-up protocol, our results are mainly based on retrospective anamnestic data collected by submitting a specifically designed questionnaire to children’s parents during follow-up appointments. Furthermore, an important limitation was the time frame of the overlapping SARS-CoV-2 pandemic, which influenced the number of infants evaluated at follow-up in the 2 groups and the spread of respiratory pathogens, considering the national lockdown ordered in Italy in March 2020: free movements within the nation were restored only in June 2020. Moreover, new restrictions were reintroduced in October 2020 to fight the so-called second and third waves of virus spread.^20^ However, the 2 groups at follow-up were representative of the initial RCT neonatal population and were comparable in terms of baseline neonatal characteristics. An important limitation is the lack of nutritional data about the administration of breastmilk vs formula milk, considering the protective effect of human milk against the risk of BPD.^21^ Additionally, an important limitation was that objective assessments of lung volumes at birth and at cPNA 2 years were not performed. Such measures of lung function could demonstrate the superiority of the IN-REC-SUR-E technique over IN-SUR-E over the long term. The study team will include lung function tests for planned follow-up of the whole multicenter population of the IN-REC-SUR-E vs IN-SUR-E RCT that will enroll children aged 5 years and older.

## Conclusions

To our knowledge, this RCT is the first study evaluating long-term outcomes in infants treated with IN-REC-SUR-E vs standard care. The results of our analysis are promising and support the safety of the IN-REC-SUR-E technique in all the studied domains. At the 2-year follow-up, there were no differences between groups in death, neurodevelopmental outcomes, anthropometric measurements, or recurrent respiratory infections. Our findings cannot be compared with data from other cohorts yet. Further multicenter studies could be helpful to better confirm our results. Neurodevelopmental outcomes, such as growth and respiratory outcomes, of all patients treated with this new procedure should be prospectively evaluated to further validate the safety and superiority of IN-REC-SUR-E compared with the standard IN-SUR-E.

## Notes

Supplement 1.
Trial Protocol and Statistical Analysis Plan

Supplement 2. **eFigure. **Mean Developmental Quotient at 24 Months’ CA in the 2 Groups

Supplement 3.
Data Sharing Statement
