# Supplementary material for: Lung Recruitment Before Surfactant Administration in Extremely Preterm Neonates: 2-Year Follow-Up of a Randomized Clinical Trial
Source: JAMA Netw Open. 2024 Sep 25;7(9):e2435347. doi: 10.1001/jamanetworkopen.2024.35347 (PMC11425149; doi:10.1001/jamanetworkopen.2024.35347)
Supplement: Supplement 1. — Trial Protocol and Statistical Analysis Plan [file jamanetwopen-e2435347-s001.pdf]

The U.S. government does not review or approve the safety and science of all studies listed on this website.

Read our full disclaimer (https://clinicaltrials.gov/about-site/disclaimer) for details.

COMPLETED

Efficacy of a New Technique -"IN-REC-SUR-E"- in Preterm Neonates With RDS

ClinicalTrials.gov ID NCT02482766

Sponsor Catholic University of the Sacred Heart

Information provided by Giovanni VENTO, Catholic University of the Sacred Heart (Responsible Party)

Last Update Posted 2019-02-15

Study Details Tab

Study Overview

Brief Summary

The primary hypothesis of this study is reduction in need of mechanical ventilation in the first 72 hours of life (excluding the transient tracheal intubation performed for surfactant administration and the mechanical ventilation for lung recruitment) in spontaneously breathing infants born at 24+0-27+6 weeks' gestation and failing nCPAP during the first 24 hours of life who received an HFOV recruitment maneuver (IN-REC-SUR-E) compared to no recruitment maneuver (IN-SUR-E) just prior to surfactant administration followed by prompt extubation.

Detailed Description

EFFICACY OF A NEW TECHNIQUE -"IN-REC-SUR-E"- IN PRETERM NEONATES WITH RESPIRATORY DISTRESS SYNDROME DURING NON INVASIVE VENTILATION: A RANDOMIZED CONTROLLED TRIAL

BACKGROUND The initial stabilization on Continuous Positive Airway Pressure (CPAP) and provision of rescue surfactant only when necessary is at least as beneficial and quite possibly preferred over the standard therapy of intubation of all infants at risk in the delivery room and subsequent support with mechanical ventilation (2-4). The percentage of CPAP failure in the newborns of 25-28 weeks' gestation is 45 % (30/66) in the Australian experience (5), higher than that reported by Ammari et al.

Feedback

(25 %) (6) and similar to that reported by De Jaegere et al. (50 %) (7) and in the COIN trial (46 %) (2). As a potential alternative, the INSURE (INtubate, SURfactant, Extubate) approach (8) is very attractive. Recently, several studies have investigated the effectiveness of the association between non-invasive ventilation and surfactant, administered by transient intubation (INSURE), showing that it is effective in preventing the need for mechanical ventilation. Although beneficial in clinical practice, the INSURE method cannot be universally applied to all preterm neonates with Respiratory Distress Syndrome (RDS) and is unsuccessful in a particular section of this population. The INSURE failure rate recently reported in preterm infants of different gestational age widely ranges from 19 % to 69 % (10, 11). Unfortunately, no randomized controlled trials have directly evaluated the efficacy of INSURE in extremely preterm neonates (<28 weeks gestation) (8). Nevertheless, the data of the "Sustained Lung Inflation (SLI) study" (14) recently conducted on infants with GA 25+0- 28+6 weeks showed that nasal CPAP failure and need for Mechanical Ventilation (MV) at 72 hours of life was 53 % in the SLI group and 65 % in the control group (only nasal CPAP). In this study surfactant was preferably administered with the INSURE approach at a Fraction of Inspired Oxygen (FiO2) threshold of 0.40 and then it could be reasonably argued that at least 50 % of 25-28 weeks' gestation infants, even if receiving a SLI maneuver and the INSURE treatment for CPAP failure, undergoes mechanical ventilation in the first 72 hours of life for unsuccessful INSURE. One of the possible mechanisms responsible for the unsuccessful INSURE, requiring subsequent re-intubation and mechanical ventilation is the inability of the preterm lung with RDS of achieving and maintaining an "optimal" Functional Residual Capacity (FRC). Prophylactic or early rescue surfactant administration before alveolar recruitment probably results in an uneven surfactant distribution to already open alveoli thus resulting in poor clinical response to the first surfactant treatment. We, therefore, seek to compare the application of a recruitment manoeuver - in High-Frequency Oscillatory Ventilation (HFOV) modality - just before the surfactant administration, followed by rapid extubation (INtubate-RECruited-SURfactant-Extubation: IN-REC-SUR-E) with INSURE alone in spontaneously breathing preterm infants requiring nasal CPAP as initial respiratory support and reaching pre-defined CPAP failure criteria, for evaluating its effectiveness in decreasing the need of MV and improving respiratory outcome.

Study design This will be an unblinded multi-center randomized trial of IN-REC-SUR-E vs. IN-SUR-E in infants born at 24+0-27+6 weeks' gestation.

Sample size The investigators hypothesized that a recruitment manoeuver (in HFOV modality) before surfactant administration might decrease of the need of subsequent mechanical ventilation during the first 72 hours of life from 50 % (2, 5, 7) to 30 %. The investigators calculated that 103 newborns must be enrolled in each group to detect this difference as statistically significant with 80 % power at 0.05 level.

Randomisation Infants at each unit will be block (1st block: gestational age from 24+0 to 25+6 weeks; 2nd block: gestational age from 26+0 to 27+6 weeks) randomly assigned to a treatment group using automatically generated sealed envelopes which will be prepared at Policlinico A. Gemelli Hospital in Rome and then distributed to participating centers. Permutated block randomization with 1:1 randomization between the two arms will be used.

Blinding The study will not be blinded, and the staff performing the study also will take care of the infants later on. However, the decision to start mechanical ventilation will be made by clinicians other than the investigators and, moreover, parents, nurses involved in patient care and researchers

assessing study end-points will be blinded to the nature of the study treatments. To minimize bias, strict criteria and definitions will be maintained during the trial.

Management in the Delivery Room Positive pressure with a neonatal mask and a T-piece system (Neopuff Infant Resuscitator, Fisher and Paykel, Auckland, New Zealand) will be used to stabilize newborns after birth. All the neonates will receive one (or two) SLI manoeuver(s) (25 cmH2O for 10-15 seconds) (14) and will be transferred to the Neonatal Intensive Care Unit (NICU) in nasal CPAP (6 cmH2O). Infants will start mechanical ventilation in agreement with the American Academy of Paediatrics guidelines on neonatal resuscitation (16). In this latter case the babies will be excluded from the study (see exclusion criteria section, page 4).

CPAP Failure Criteria In the NICU, nasal CPAP will be given through nasal prongs/mask using the standard method of the single centre (ventilator, flow-dependent system) with an initial pressure of 6 to 7 cmH2O, in all infants. CPAP failure is defined if they met any of the following criteria: FiO2 ≥ 0.30 on nasal CPAP (17) to maintain pulse oximetry (SpO2) 87-94 %(18) for at least 30 minutes unless rapid clinical deterioration occurred, respiratory acidosis defined as pCO2 > 65 mmHg (8.5 kPa) and pH < 7.20 on arterial or capillary blood gas sample, apnea defined as >4 episodes of apnea per hour or >2 episodes of apnea per hour when ventilation with bag and mask will be required.

HFOV Recruitment manoeuver Infants in the IN-REC-SUR-E group will undergo the following approach: after intubation, HFOV will be delivered with the ventilator available in each NICU. The following initial ventilator setting will be advocated: Continuous Distending Pressure (CDP): 8cmH2O; Frequency: 10-15 Hz; Delta P: 15 cmH2O or Amplitude 30 % eventually increased - chest to be "visibly vibrating "-; I:E 1:2. Delta P (or amplitude) first and/or frequency subsequently will be adjusted to achieve a Tidal Volume (VT) of 1,5-2 ml/kg and/or to maintain the transcutaneous partial carbon dioxide pressure (TcPCO2) between 40 and 60 mmHg (5.3 and 8.0 kPa). The infants will be subjected to an open lung ventilation strategy aiming to recruit and stabilize the majority of collapsed alveoli/sacculi, using oxygenation as an indirect parameter for lung volume. Optimal recruitment is defined as adequate oxygenation using a FiO2 of 0.25 or less. Starting at 8 cmH2O, the CDP will be increased stepwise (2 cmH2O every 2-3 minutes) as long as SpO2 improves. The FiO2 will be reduced stepwise, keeping SpO2 within the target range (87-94 %). The recruitment procedure will be stopped if oxygenation no longer improves or if the FiO2 is equal to or less than 0.25. The corresponding CDP will be called the opening pressure (CDPO). Next, the CDP will be reduced stepwise (1-2 cmH2O every 2-3 minutes) until the SpO2 deteriorates (of at least 2-3 points). The corresponding CDP will be called the closing pressure (CDPC). After a second recruitment manoeuver at CDPO for 2 minutes, the optimal CDP (CDPOPT) will be set 2 cmH2O above the CDPC for at least 3 minutes (19). A chest radiograph at this point is advised.

Surfactant Treatment

Infants in the IN-REC-SUR-E arm will undergo the following approach: as soon as possible after the recruitment manoeuver (at CDPOPT) a dose of poractant alfa (Curosurf [Chiesi Farmaceutici, Parma, Italy]) of 200 mg/kg will be administered via a closed administration system in one-two aliquots (1-2 minutes). The tube position will be confirmed by auscultation. A temporary reduction of frequency may be necessary to increase the VT up to 2.5 ml/kg for improving the surfactant spreading.

Infants in the IN-SUR-E arm will undergo the following approach: after intubation, a dose of poractant alfa (Curosurf [Chiesi Farmaceutici, Parma, Italy]) of 200 mg/kg will be administered via a closed administration system in one-two aliquots (1-2 minutes). The tube position will be confirmed

by auscultation. During surfactant administration, infants will be manually ventilated to facilitate surfactant distribution. If necessary, mechanical ventilation with a peak inspiratory pressure (PIP) of 20-22 cmH2O, a Positive End-Expiratory Pressure (PEEP) of 5-6 cmH2O and a respiratory rate of 30-40 breaths/min will be subsequently started to achieve a VT of 4-6 ml/kg and/or to maintain the transcutaneous partial carbon dioxide pressure (TcPCO2) between 40 and 60 mmHg (5.3 and 8.0 kPa).

After surfactant administration, the babies of both groups will be extubated within 30 minutes (if satisfactory respiratory drive is present) and will receive nasal CPAP (6-8 cmH2O) (20). In case of insufficient respiratory drive, CDP (in the babies of IN-REC-SUR-E arm) or PIP (in the IN-SUR-E arm) will be reduced until spontaneous respiratory activity is restored. Maintaining a FiO2 < 0.30 to obtain SpO2 values in the desired range (87-94 %) will drive the eventual reduction in the level of CPAP in the following days. The decision as to whether to begin Bi-level Positive Airway Pressure (BiPAP) or nasal-Intermittent Mandatory Ventilation (N-IMV) to prevent the need for re-intubation in infants of both groups will be up to the neonatologist on duty, and will be considered in the final analysis.

Infants of both groups can receive a subsequent dose of surfactant (100 mg/kg of poractant alfa) using the same method (INSURE or INRECSURE) if they meet the CPAP failure criteria again during the following 12 to 24 hours.

Data collection All collected data can be obtained from the clinical records. They will be reported in electronic data sheets designed for this study.

Statistical analysis The primary efficacy analysis will be conducted on an intention to treat basis. Clinical characteristics of infants in the "IN-REC-SUR-E" and "IN-SUR-E" groups will be described using mean values and standard deviation, median value and range, or rate and percentage. Univariate statistical analysis will be performed using the Student "t" test for parametric continuous variables, the Wilcoxon rank-sum test for non-parametric continuous variables, and Fisher's exact test for categorical variables. A $p < 0.05$ will be considered statistically significant. Then, "IN-REC-SUR-E" treatment and clinical characteristics which are most likely associated with the need for mechanical ventilation (gestational age, birth weight, antenatal steroids, CRIB score) will be included in multiple logistic regression analysis to assess their independent role in predicting "IN-REC-SUR-E" success or failure. Effect estimates will be expressed as relative risk (RR) with profile likelihood-based 95 % confidence limits.

An interim analysis is planned when 50 infants will be enrolled in each arm. Duration of study: 1 year

QUALITY CONTROL AND QUALITY ASSURANCE PROCEDURES Compliance to protocol Compliance will be defined as full adherence to protocol. Compliance with the protocol will be ensured by a number of procedures as described below.

Site set-up Local principal investigators are required to participate in preparatory meetings in which details of study protocol, data collection, "IN-REC-SUR-E" and "IN-SUR-E" procedures will be accurately discussed. All centers will receive detailed written instruction on web based data recording, and, to solve possible difficulties, it will be possible to contact the Clinical Trials Coordinating Center. Moreover, it has been ascertained that "IN-REC-SUR-E" procedure is followed similarly in all participating centers.

Official Title

Efficacy of a New Technique -"IN-REC-SUR-E"- in Preterm Neonates With Respiratory Distress Syndrome During Non Invasive Ventilation: a Randomized Controlled Trial

Conditions

Respiratory Distress Syndrome   Hyaline Membrane Disease

Intervention / Treatment

- Drug: Poractant alfa, 200 mg/kg
- Device: Ventilator for High-frequency Oscillatory Ventilation (HFOV)
- Device: Nasal Continuous Positive Airway Pressure (nCPAP)

Other Study ID Numbers

- Prot. rs 25882/14

Study Start

2015-11

Primary Completion (Actual)

2018-09

Study Completion (Actual)

2018-09-30

Enrollment (Actual)

206

Study Type

Interventional

Phase

Not Applicable

Resource links provided by the National Library of Medicine

Genetic and Rare Diseases Information Center (https://rarediseases.info.nih.gov/gard) resources: Respiratory Distress Syndrome, Infant (https://rarediseases.info.nih.gov/diseases/112/respiratory-distress-syndrome-infant) Acute Respiratory Distress Syndrome (https://rarediseases.info.nih.gov/diseases/5698/acute-respiratory-distress-syndrome)

Drug Information (https://dailymed.nlm.nih.gov/dailymed/) available for: Poractant alfa (https://dailymed.nlm.nih.gov/dailymed/search.cfm?labeltype=human&query=Poractant+alfa)

Other U.S. FDA Resources (https://classic.clinicaltrials.gov/ct2/info/fdalinks)

Contacts and Locations

This section provides the contact details for those conducting the study, and information on where this study is being conducted.

Italy

Alessandria, Italy
SS Antonio e Biagio e Cesare Arrigo

Ferrara, Italy
Azienda Ospedaliera Universitaria Ferrara

Firenze, Italy
Careggi

Foggia, Italy
Ospedali Riuniti

L'Aquila, Italy
[illegible]

Click to view interactive map

Participation Criteria

Researchers look for people who fit a certain description, called eligibility criteria. Some examples of these criteria are a person's general health condition or prior treatments.

For general information about clinical research, read Learn About Studies (https://clinicaltrials.gov/study-basics/learn-about-studies).

Eligibility Criteria

Description

Inclusion Criteria:

1. In-Born at 24+0-27+6 (and)
2. Spontaneously breathing at birth but requiring respiratory support (CPAP or O2) at 5' of life (and)
3. Parental consent has been obtained (and)
4. Failing nCPAP during the first 24 hours of life

Exclusion Criteria:

1. Severe birth asphyxia or a 5-minute Apgar score <3
2. Endotracheal intubation in the delivery room for resuscitation or insufficient respiratory drive according to AAP guidelines16
3. Prolonged PROM > 3 weeks
4. Presence of major congenital malformations
5. Hydrops fetalis
6. Inherited disorders of metabolism

Ages Eligible for Study

24 Weeks to 27 Weeks (Child)

Sexes Eligible for Study

All

Accepts Healthy Volunteers

No

Study Plan

This section provides details of the study plan, including how the study is designed and what the study is measuring.

How is the study designed?

What is the study measuring?

Primary Outcome Measures

Outcome Measure	Measure Description	Time Frame
Need for mechanical ventilation within the first 3 days of life and therefore we consider IN-REC-SUR-E a success if mechanical ventilation is not required and a failure if the infant needs mechanical ventilation in the first 72 hours.		First 72 hours of life

Secondary Outcome Measures

Outcome Measure	Measure Description	Time Frame
Duration of NCPAP		Participants will be followed for the duration of hospital stay, an expected average of 12 weeks

Duration of conventional mechanical ventilation		Participants will be followed for the duration of hospital stay, an expected average of 12 weeks
Duration of High-frequency oscillatory ventilation (HFOV)		Participants will be followed for the duration of hospital stay, an expected average of 12 weeks
Duration of O2-therapy		Participants will be followed for the duration of hospital stay, an expected average of 12 weeks
Duration of hospitalization		Participants will be followed for the duration of hospital stay, an expected average of 12 weeks

Number of doses of surfactant		First 72 hours of life
Occurrence of BPD (mild, moderate and severe forms)		Participants will be followed for the duration of hospital stay, an expected average of 12 weeks
Time to be out of any respiratory support		Participants will be followed for the duration of hospital stay, an expected average of 12 weeks

Other Outcome Measures

**Outcome Measure**	**Measure Description**	**Time Frame**
Air leaks	"Mono or bilateral pneumothorax" ; "Pulmonary interstitial Emphysema"	Participants will be followed for the duration of hospital stay, an expected average of 12 weeks

Pulmonary hemorrage	Sudden onset of overt bleeding or frank evidence of blood in the airway, leading to acute respiratory distress or respiratory failure, with diffuse, bilateral pulmonary infiltrates on chest radiograph.	First 72 hours of life
PDA and need of surgical closure		Participants will be followed for the duration of hospital stay, an expected average of 12 weeks
Hour(s) of administration of surfactant dose(s)		First 72 hours of life
3°- 4° IVH		First week of life
PVL		Participants will be followed for the duration of hospital stay, an expected average of 12 weeks
>2° ROP		Participants will be followed for the duration

		of hospital stay, an expected average of 12 weeks
NEC		Participants will be followed for the duration of hospital stay, an expected average of 12 weeks
Sepsis	Sepsis is defined as a positive blood culture or suggestive clinical and laboratory findings leading to treatment with antibiotics for at least 7 days despite absence of a positive blood culture.	Participants will be followed for the duration of hospital stay, an expected average of 12 weeks
Lenght of stay in NICU		Participants will be followed for the duration of hospital stay, an expected average of 12 weeks

Use of systemic postnatal steroids	Number of cycles of postnatal steroids i.v.: "None", "1", "> 1 cycle"	Participants will be followed for the duration of hospital stay, an expected average of 12 weeks
Mortality		Participants will be followed for the duration of hospital stay, an expected average of 12 weeks

Collaborators and Investigators

This is where you will find people and organizations involved with this study.

Sponsor

Catholic University of the Sacred Heart

Collaborators

- Ospedali Riuniti Ancona
- Ospedale Careggi, Florence, Italy
- Fondazione IRCCS Ca' Granda, Ospedale Maggiore Policlinico
- Centre for Neonatal Research and Education, Crawley, West Australia
- Monash University
- Azienda Ospedaliera San Gerardo di Monza
- Academisch Medisch Centrum - Universiteit van Amsterdam (AMC-UvA)

Investigators

- Principal Investigator: Giovanni Vento, MD, Catholic University of the Sacred Heart

Publications

The person responsible for entering information about the study voluntarily provides these publications. These may be about anything related to the study.

General Publications

No publications available

*** Find Publications about Study Results and related Pubmed Publications in the "Results" section of the study record.**

Study Record Dates

These dates track the progress of study record and summary results submissions to ClinicalTrials.gov. Study records and reported results are reviewed by the National Library of Medicine (NLM) to make sure they meet specific quality control standards before being posted on the public website.

Study Registration Dates

First Submitted

2015-06-01

First Submitted that Met QC Criteria

2015-06-23

First Posted (Estimated)

2015-06-26

Study Record Updates

Last Update Submitted that met QC Criteria

2019-02-11

Last Update Posted

2019-02-15

Last Verified

2019-02

More Information

Terms related to this study

Additional Relevant MeSH Terms

Disease
Pathologic Processes
Lung Diseases
Respiratory Tract Diseases
Respiration Disorders
Infant, Premature, Diseases
Infant, Newborn, Diseases
Respiratory Distress Syndrome
Respiratory Distress Syndrome, Newborn
Hyaline Membrane Disease
Syndrome
Pulmonary Surfactants
Respiratory System Agents
Poractant alfa
