# Supplementary material for: Lung Recruitment Before Surfactant Administration in Extremely Preterm Neonates: 2-Year Follow-Up of a Randomized Clinical Trial
Source: JAMA Netw Open. 2024 Sep 25;7(9):e2435347. doi: 10.1001/jamanetworkopen.2024.35347 (PMC11425149; doi:10.1001/jamanetworkopen.2024.35347)
Supplement: Supplement 2. — eFigure. Mean Developmental Quotient at 24 Months’ CA in the 2 Groups [file jamanetwopen-e2435347-s002.pdf]

Supplemental Online Content

Gallini F, De Rose DU, Iuliano R, et al; and the IN-REC-SUR-E Study Group. Lung recruitment before surfactant administration in extremely preterm neonates: 2-year follow-up of a randomized clinical trial. *JAMA Netw Open*. 2024;7(9):e2435347. doi:10.1001/jamanetworkopen.2024.35347

eFigure. Mean Developmental Quotient at 24 Months' CA in the 2 Groups

This supplemental material has been provided by the authors to give readers additional information about their work.

© 2024 Gallini F et al. *JAMA Network Open*.

eFigure. Mean Developmental Quotient at 24 Months' CA in the 2 Groups

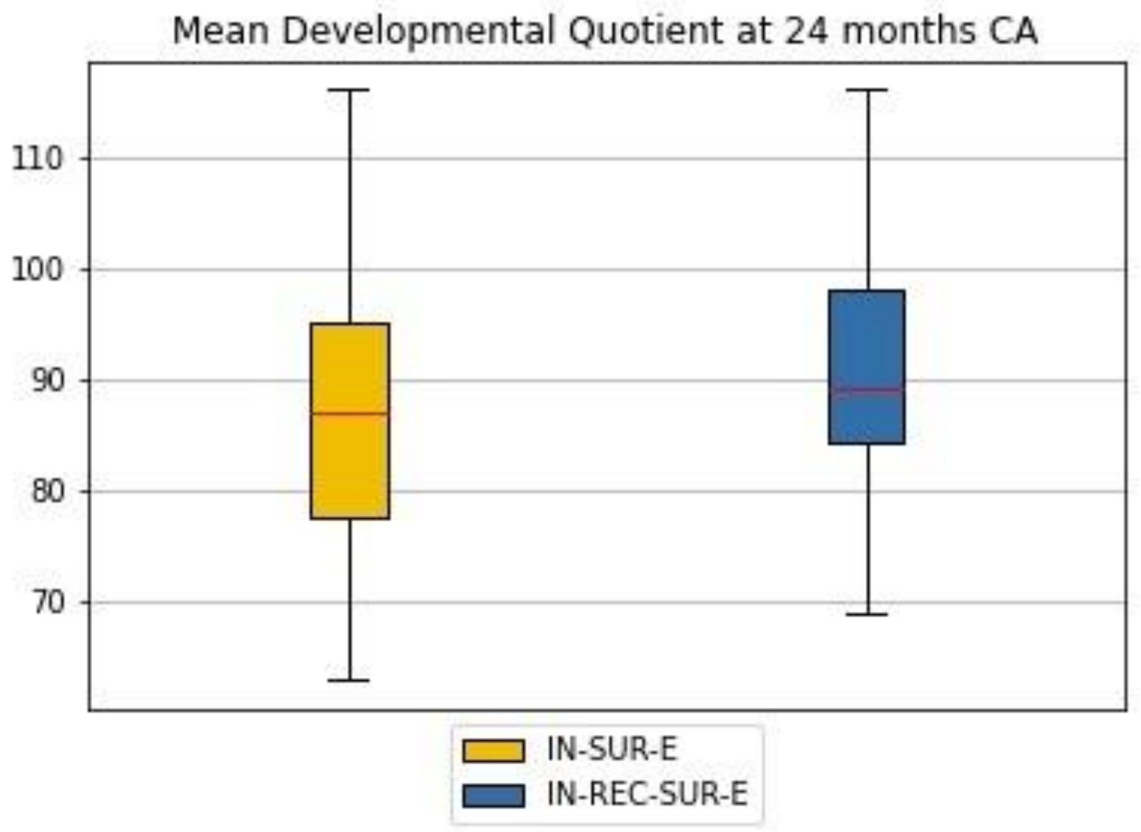

© 2024 Gallini F et al. *JAMA Network Open*.
