# Supplementary material for: Lung Recruitment Before Surfactant Administration in Extremely Preterm Neonates: 2-Year Follow-Up of a Randomized Clinical Trial
Source: JAMA Netw Open. 2024 Sep 25;7(9):e2435347. doi: 10.1001/jamanetworkopen.2024.35347 (PMC11425149; doi:10.1001/jamanetworkopen.2024.35347)
Supplement: Supplement 3. — Data Sharing Statement [file jamanetwopen-e2435347-s003.pdf]

Data Sharing Statement

Gallini. Lung Recruitment Before Surfactant Administration in Extremely Preterm Neonates. *JAMA Netw Open*. Published September 25, 2024. doi:10.1001/jamanetworkopen.2024.35347

Data
Data available: No

Additional Information
Explanation for why data not available: Data requests can be submitted at any time to the coordinator of the INRECSURE trialy, and the data will be accessible for 12 months from publication, with possible extensions considered.
